# Influence of Framework Material on Stress, Fatigue, and Stability of “All-on-Four” System Components—Biomechanical Evaluation with Finite Element Analysis

**DOI:** 10.3390/jfb17050238

**Published:** 2026-05-08

**Authors:** Dijana Popovic Grubac, Djordje Bozovic, Jelena Lecic, Ines Kovacic, Ognjenka Janjic Pavlovic, Aleksandra Zuza, Dea Krsticevic, Nedeljka Ivkovic

**Affiliations:** 1 Department of Dentistry, Faculty of Medicine Foca, University of East Sarajevo, 73300 Foca, Bosnia and Herzegovina; 2 Department of Removable Prosthodontics, University of Zagreb School of Dental Medicine, 10000 Zagreb, Croatia; 3 Independent Researcher, 88000 Mostar, Bosnia and Herzegovina

**Keywords:** all-on-four, finite element analysis, framework material, stress distribution, material fatigue

## Abstract

The “all-on-four” concept is a prominent solution for rehabilitating edentulous patients with fixed full-arch restorations. This study aimed to examine the stress distribution pattern, material fatigue, and restoration displacement using different framework materials under static (SL) and dynamic (DL) loading conditions. Six three-dimensional finite element analysis models of an atrophic maxilla rehabilitated via the all-on-four concept were analyzed. Models utilized three different framework materials: Cobalt–Chromium Alloy (CoCr), Zirconia (Zr), and Polyetheretherketone (PEEK). The models were subjected to three types of SL (90, 150, and 200 N) and two cases of DL (150 N) simulating mastication. DL generated higher stress intensities compared to SL. PEEK models showed the highest stress concentrations in the cortical bone (up to 70.44 MPa) and implants across SL models. The PEEK frameworks showed a risk of fatigue-related fracture of the cortical bone around terminal implants. Models utilizing PEEK frameworks exhibited significantly greater structural displacement (up to −0.681 mm horizontally) under DL compared to their rigid counterparts. CoCr and Zr provide better resistance to cyclic loading and reduced displacement, ensuring a higher safety factor. PEEK frameworks demonstrated inferior mechanical resistance under fatigue.

## 1. Introduction

Tooth loss is a significant problem for humanity, and it is caused by various factors such as caries, periodontitis, and trauma. The conventional treatment option for edentulous patients is the use of removable complete dentures. Implant prosthetic therapy for edentulous jaws offers patients the possibility of rehabilitation with full-arch fixed restorations, which improve retention, stabilization, and overall quality of life. The leading concept in recent years is the concept of “all-on-four” implants [1,2,3,4,5]. The advantages of this concept are reflected in the avoidance of complicated surgical interventions, the insertion of only four implants in an atrophic jaw, and the possibility of immediate fabrication of temporary restorations [6]. The patient receives a definitive restoration after the period of osseointegration. Fixed full arch restorations on implants in the “all-on-four” concept can be made by conventional laboratory procedures, and computer-aided design and computer-aided manufacturing (CAD-CAM). Different materials are used for its manufacture, including metal alloys, ceramics, and polymer materials [7]. The clinician is often faced with the dilemma of choosing the right material. The choice of restorative material is influenced by numerous factors, such as the inter-arch space, i.e., the available space for the restoration, as well as economic reasons, the presence of allergies to some restorative materials, the presence of parafunctions, and others [8]. Also, the fatigue of implant-supported restorations is important for the durability and safety of the therapy. Analyzing all of the above allows dentists to choose optimal materials and designs, prevent complications, and ensure that implants last as long as possible [8].

Numerous studies [1,9,10,11,12,13,14,15] have established that the framework material modulus of elasticity affects the stress distribution on the alveolar bone, implant, and prosthetic components. Restorations whose framework has a higher modulus of elasticity under the influence of load absorb more stress and transfer less stress to the bone around the implant. On the contrary, frameworks with a lower modulus of elasticity transmit more stress to the bone and contribute to a greater load on the implant. Most of the available studies investigated the influence of static loading on stress distribution. Their design differs in force values and force application point [12,13,14,16,17,18,19].

However, dynamic loading is a more faithful representation of mastication. It generates forces that are about 20 percent greater than static ones [8,20]. The forces generated in the oral cavity by the masticatory muscles are transmitted through the occlusal complex to the implants and the surrounding bone. These forces induce stresses. Stress distribution in implant prosthetics is a key factor affecting the durability and success of the implants. Specifically, unequal force distribution can lead to overloading of the implants, which may result in bone loss, implant mobility, and ultimately, implant failure [1]. Therefore, understanding how different forces affect stress distribution is crucial for planning and executing implant prosthetic therapy [21,22]. One of the leading experimental methods for studying stress distribution is the finite element analysis (FEA). Computer simulations on the model illustrate the force distribution under various load conditions, thereby enabling optimization of implant position and prosthetic design [1].

This study aims to examine the stress distribution patterns in various restoration materials under static and dynamic loading, as well as under material fatigue and restoration displacement.

## 2. Materials and Methods

### 2.1. Experimental Design

A completely edentulous maxilla was designed to simulate an atrophic maxilla with moderate sinus pneumatization that would be rehabilitated with a full fixed restoration according to the all-on-four concept.

In this study, three framework materials were used (CoCr, Zr, and PEEK), and for each of these two types of veneers, i.e., composite (Gradia) and ceramic (Feldspathic ceramic). There were 6 models in total (Table 1).

### 2.2. Modeling

A 3D model of an edentulous upper jaw fabricated from artificial resin was scanned using an intraoral scanner (3Shape, Copenhagen, Denmark). The same scanner was used to scan the implants and their components. In the present study, Zimmer implants (Zimmer Biomet, Warsaw, IN, USA) with a diameter of 4.1 mm and a length of 11.5 mm for the anterior ones and 13 mm for the posterior ones were used. The two anterior implants were inserted in the edentulous jaw model vertically in the area of the lateral incisors and the posterior ones in the area of the second premolars, in front of the anterior wall of the maxillary sinus. The lateral implants were angled at 30° [18]. Multiunit abutments were placed on the implants. The anterior ones were straight (2.5 mm), and the lateral ones were angled, measuring 3.5 mm in height. The implant–abutment connection was an internal hexagon. The screws were modeled in accordance with the Zimmer Biomet USA catalog (catalog No. SCTS).

The implants were inserted in the edentulous jaw model; abutments were placed, and the entire assembly was scanned.

In CAD (computer-aided design) software (Geomagic Design X, 3D Systems, Rock Hill, SC, USA), a restoration has been scanned into the model. The restoration framework was 12 mm high. The crowns were taken from the computer database.

All scanned models (jaw, implants, implant components, restoration parts) were exported in STL format. Then, using reverse-engineering software, they were converted into a three-dimensional CAD model. The Geomagic Design X program (USA) was used to reconstruct the model geometry. The model looked like this: A 1 mm layer of cortical bone surrounded the cancellous bone, corresponding to D3 bone density [3,21,23]. The pneumatization of the maxillary sinus was present. The height of the alveolar ridge was 12 mm, which corresponds to pronounced jaw atrophy. The mucosa was excluded from the model due to its minimal effect on load distribution [18]. The prosthetic component was a fixed restoration retained with screws. It consisted of a framework and crowns. The crowns were 2 mm thick. The restoration material was different: feldspathic ceramic and Gradia composite. The cantilever length was constant at 10 mm [19,24].

### 2.3. Meshing Procedure

**Figure 1:**
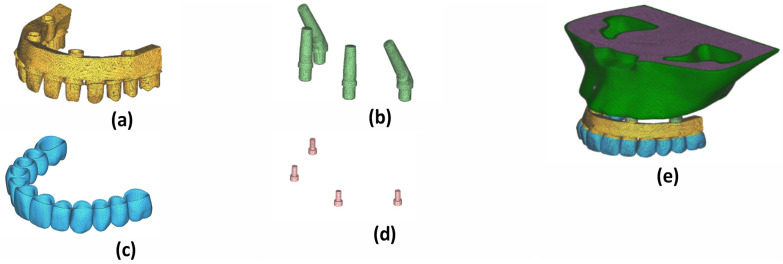
Finite element model with mesh structure: (**a**) framework, (**b**) Implant abutment complex, (**c**) crowns, (**d**) screw, (**e**) definitive maxillary model.

All geometric models were then exported to a STL file and combined to create a finite element model using Femap version 2021.2 (Siemens PLM Software, Plano, TX, USA). All components of the model are assumed to be homogeneous, isotropic, and linearly elastic. Tetrahedral elements and nodes were used to create the finite element mesh (Figure 1). The number of nodes and mesh density were presented in Table 2.

### 2.4. Boundary and Loading Conditions

The mechanical properties of the material are presented in terms of Young’s modulus of elasticity, Poisson’s ratio, and density (Table 3). The model of the maxilla was fixed to the base of the skull, with zero degrees of freedom. The connection of the implant and the alveolar bone simulates complete osseointegration. Trabecular–cortical bone, implant–bone, implant–abutment, abutment–framework, and framework–crown; all these contacts are considered to be bonded [11].

#### 2.4.1. Loading Conditions

##### Static Loading

**Figure 2:**
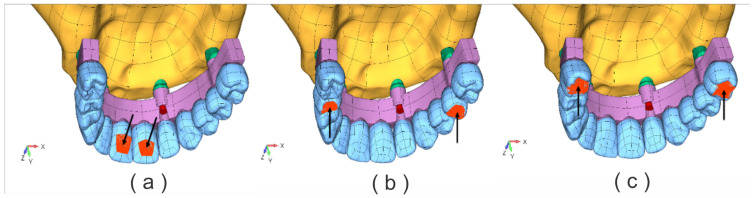
Applied occlusal forces; (**a**) SL1—horizontal direction F = 90 N; (**b**) SL2—vertical direction. F = 150 N; (**c**) SL3—vertical direction F = 200 N.

In this study, three types of occlusal static loads were applied to all models [20], as shown in Figure 2:

- A horizontal static load (SL1) of 90 N was applied on the palatal surface of the central incisors (Figure 2a).
- A bilateral vertical static load (SL2) of 150 N was applied to the occlusal surface of the second premolar (Figure 2b).
- A bilateral vertical static load (SL3) of 200 N was applied to the occlusal surface of the first molar (Figure 2c).

##### Dynamic Loading

**Figure 3:**
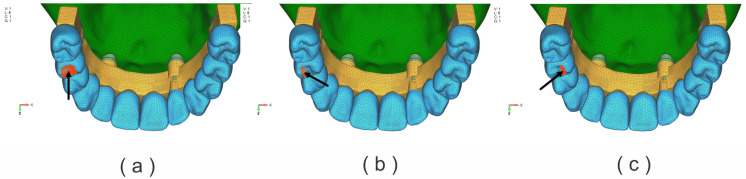
Applied dynamic loads on the second left premolar (D5): (**a**) vertical direction; (**b**) oblique direction, palatal slope of the buccal cusp; (**c**) oblique direction, buccal slope of the palatal cusp.

**Figure 4:**
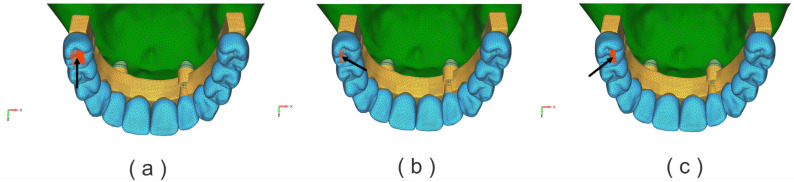
Applied dynamic loads on the first left molar (D6): (**a**) vertical direction; (**b**) oblique direction, palatal slope of the buccal cusp; (**c**) oblique direction, buccal slope of the palatal cusp.

Dynamic analysis of the structure was performed as described in the literature [25]. Two cases of dynamic loading (DL) were analyzed, namely:

- variable dynamic loading on the left upper second premolar (D5), with a maximum intensity of 150 N (Figure 3a–c);
- variable dynamic loading on the left upper first molar (D6), with a maximum intensity of 150 N (Figure 4a–c);

The bite forces were applied dynamically to more accurately mimic the chewing process. A chewing force of 150 N was set as a working lateral posterior tooth load. A dynamic load period of 0.875 s was set, and the left side of the lateral movement was set as the working side. The dynamic load acted in five different phases [25]:

Loading stage 1—no load; loading time 0.00–0.13 s;

Loading stage 2—loading time 0.13–0.15 s; force acts perpendicular to the occlusal plane; position buccal tip, and tongue tip position;

Loading stage 3—loading time 0.15–2.60 s; force acts from the lingual side to the buccal side with 45º; position lingual slopes of the buccal tips;

Loading stage 4—loading time 0.26–0.30 s; from the buccal side to the lingual side with 45º; position buccal slopes of the lingual tips;

Loading stage 5—no load; loading time 0.3–0.875 s; (Figure 3 and Figure 4).

The maximum load intensity was the same across stages, although the loading positions and directions differed.

### 2.5. Stress Analysis

Von Mises stress was used for stress analysis. Stress values were automatically calculated in MPa using the software’s range, color, and magnitude scales.

#### 2.5.1. Fatigue Analysis

The fatigue assessment was conducted based on the maximum stress values derived from dynamic analysis under variable loading conditions at the second left premolar and first left molar. The evaluation considered the material combinations presented in Table 1.

The maximum stress values that occurred in each of the materials for the analyzed load cases were compared with the Wöhler fatigue curve, SN curve taken from the literature [20,26]. In the case when the value of the maximum stress in the material is below the SN curve of the corresponding material at the maximum number of cycles (10^10^), it is said that the permanent dynamic strength of the material is satisfied, and it is concluded that no fatigue fracture will occur in the material. In the case when this condition is not met and when the line representing the maximum stress value intersects the SN curve of the corresponding material, it is said that the permanent dynamic strength is not satisfied. Based on the intersection point of these two curves, the number of load cycles at which fatigue fracture will occur in the material was determined. In this way, the service life of the structure can be determined.

#### 2.5.2. Displacement

The displacement of restorations under occlusal loads is presented in micrometers and is shown in both horizontal and vertical directions. The displacement values are presented in tabular form for the horizontal and vertical directions.

Displacements, stress, and fatigue analyses were calculated for each component (bone, implant/abutments, framework, and veneering) under applied forces.

## 3. Results

### 3.1. Static Loading

**Figure 5:**
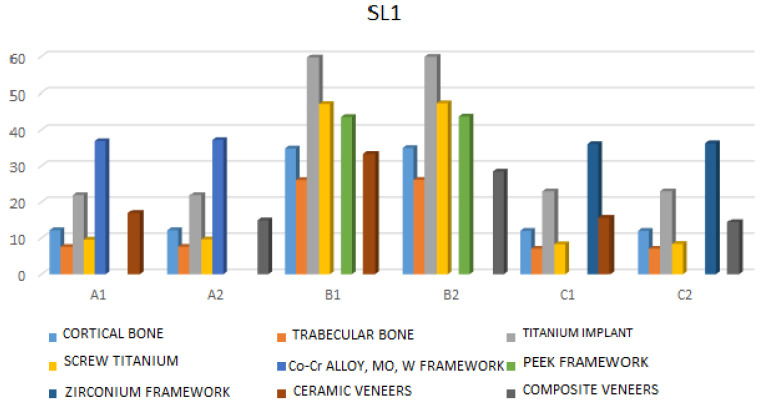
Maximum stress values under horizontal static loadings (SL1) (F = 90 N, teeth: right central incisor, left central incisor).

SL1—Under this type of loading, the highest stress values are observed in the following: cortical bone in model B2—which consists of a PEEK framework and composite veneers, (34.96 MPa), in the projection of the neck of the front implants; cancellous bone in model B2 (26.17 MPa) in the neck projection of the front implants; and implant stress is also highest in model B2 (59.90 MPa) in the region of the implant neck and at the junction with the abutment. The highest stress on the framework is found in model B2 (43.98 MPa) in the projections of the abutment position of the anterior implants; the highest stress on the screw is also in model B2 (47.38 MPa) on the surface of the front screws in the area of force action; while the highest stress on the veneering material is recorded in model B1, which has a PEEK framework and ceramic veneers, (33.33 MPa), on the cervical edge of the crown in the area of force action (Figure 5 and Figures S1–S7).

With SL2 load type B (B1 and B2), the models show the highest stress value on cortical bone, implant spongiosa and screws. Framework and veneers show less stress in B models compared to A and C.

During this load, models with a PEEK framework retain less stress in the framework and veneers, while greater stress is transferred to other structures.

**Figure 6:**
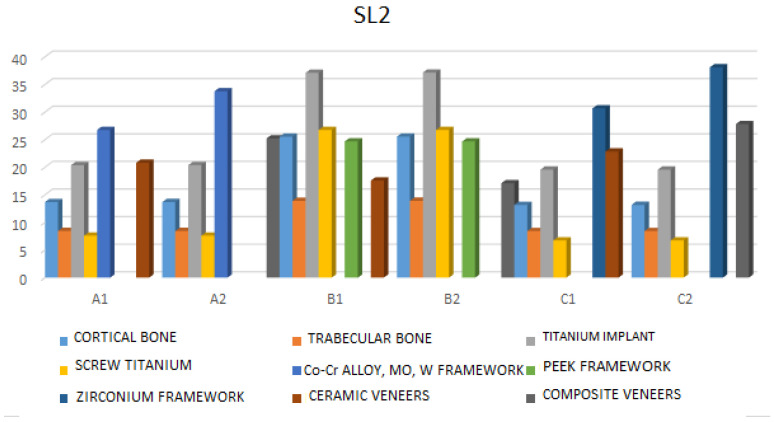
Maximum stress values under bilateral vertical static loadings (SL2) (F = 150 N, teeth: right central incisor, left central incisor).

SL2—Under this type of loading, the highest stress values are observed in the cortical bone in model B2 (25.54 MPa), cancellous bone in model B2 (13.93 MPa), and the highest implant stress is also in model B2 (37.15 MPa). Therefore, B models (B1 and B2) exhibit the highest stress values in cortical bone, implant spongiosa, and screws. During this load, models with a PEEK framework retain less stress in the framework and veneers, while greater stress is transferred to other structures. Framework and veneers show less stress in B models compared to A and C. The highest stress on the framework is found in model C2 (38.11 MPa), the highest stress on the screw is in models B1 and B2 (21.76 MPa), and the highest stress on the veneering material is recorded in model C1 (28.85 MPa) (Figure 6 and Figures S2–S8).

SL3—Under this type of loading, the highest stress values are observed in the cortical bone in model B2 (70.44 MPa), cancellous bone in model B2 (49.67 MPa), and the highest implant stress is also in model B2 (92.99 MPa).

The highest stress on the framework is found in model C1 (103.06 MPa), the highest stress on the screw is in model B2 (60.20 MPa), and the highest stress on the veneering material is recorded in model B1 (69.29 MPa) (Figure 7, Figures S2–S7 and S9).

### 3.2. Dynamic Loading

The results are presented for cases when dynamic loading is applied to teeth 25 and 26.

**Figure 8:**
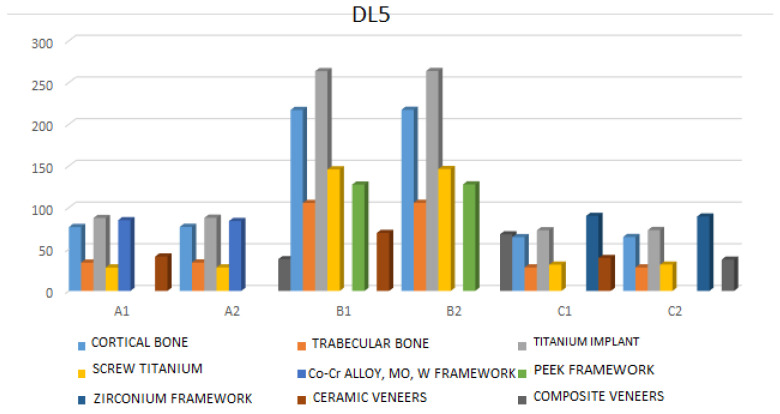
Maximum stress values under dynamic loadings (F = 150 N, tooth: the second left premolar).

DL5—The maximum value of Von Mises stress in the cortical bone is observed in model B1 (149.96 MPa). In the trabecular bone, the highest von Mises stress for the same material is 77.20 MPa. For implants, the maximum von Mises stress occurs in model B2 (180.24 MPa), and a similar situation is observed for the screws in model B2 (102.92 MPa). The framework showing the highest von Mises stress is model A2 (94.97 MPa), while the highest stress is retained in the ceramic crowns of model B1 (93.23 MPa) (Figure 8 and Figures S2–S7).

**Figure 9:**
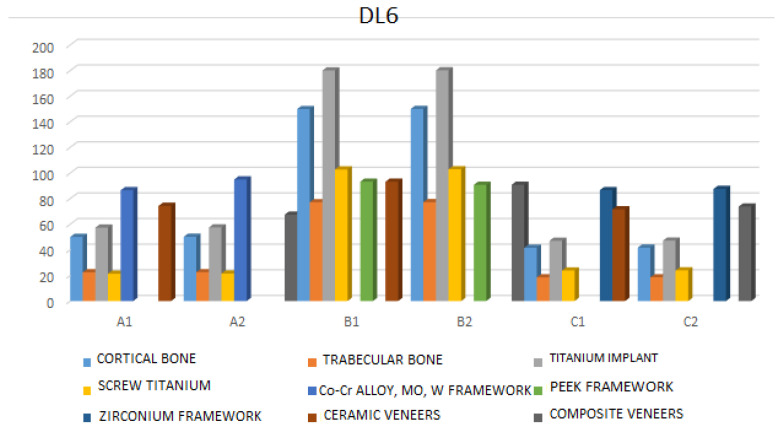
Maximum stress values under dynamic loadings (F = 150 N, tooth: the first left molar).

DL6—The cortical bone exhibits the highest von Mises stress in case B2 (217.25 MPa), and the cancellous bone shows the highest stress in case B2 (105.66 MPa). The maximum von Mises stress for implants and screws is observed in model B2 (263.79 MPa and 146.23 MPa, respectively). The highest von Mises stress in the framework under this loading condition is found in the PEEK B2 model (127.79 MPa), while the crowns in model B1 exhibit the maximum von Mises stress value (67.88 MPa) (Figure 9, Figures S2–S7 and S10).

### 3.3. Material Fatigue Results

**Figure 10:**
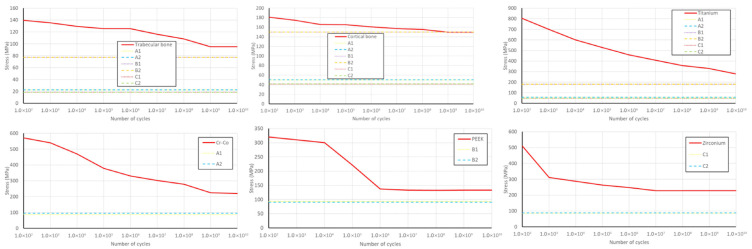
S–N curves for different components of the system at the position of the second left premolar.

**Figure 11:**
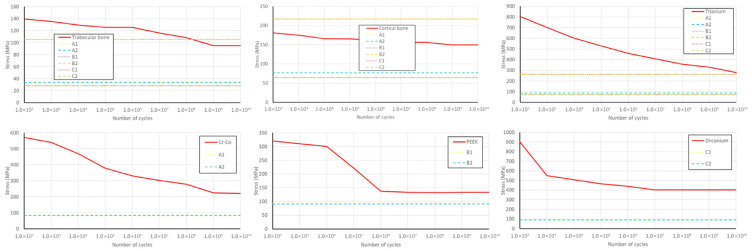
S–N curves for different components of the system at the position of the first left molar.

Material fatigue is indicated by the relationship between the maximum stress value and the S–N curve for specific materials, as observed in the literature (Figure 10 and Figure 11).

The results indicate that the permanent dynamic strength was not met for B1 and B2 materials.

### 3.4. Displacement Results

**Figure 12:**
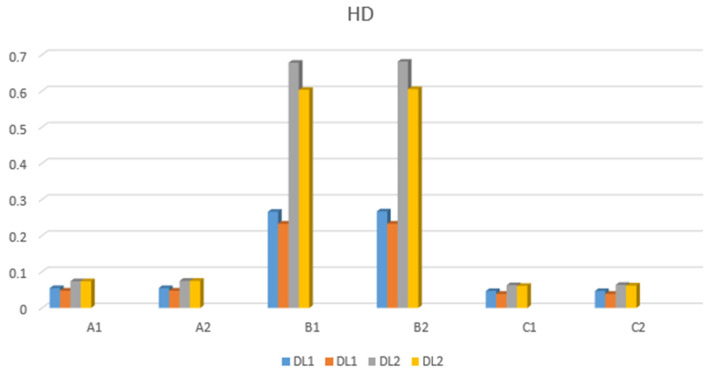
Horizontal displacement values.

**Figure 13:**
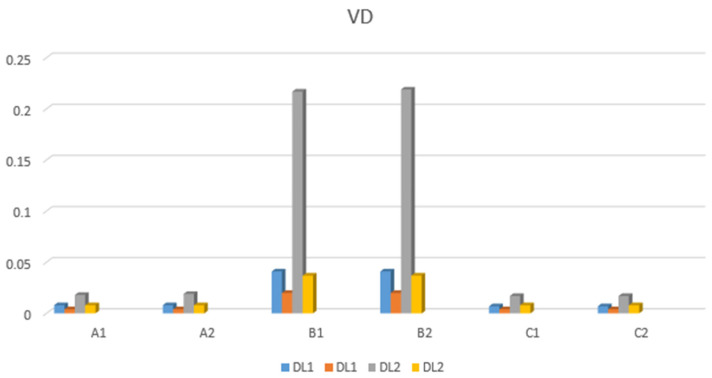
Vertical displacement values.

The displacement of the restoration under dynamic loading is summarized in Figure 12 and Figure 13, which present the results for loading applied to the second left premolar (D5) and the first left molar (D6).

#### 3.4.1. Horizontal Direction

Figure 12 shows that the highest minimum displacement value when loading on D5 is for model B2 (0.267 mm), while the maximum displacement value for models A1 and A2 is 0.048 mm.

When the load acts on tooth D6, the highest minimum displacement value is for model B1 (=0.681 mm), while the maximum displacement value (0.605 mm) is for B2 (Figure 12).

#### 3.4.2. Vertical Direction

The largest displacement during tooth loading was observed in models B1 and B2 (0.041 mm), with a maximum displacement value of 0.020 mm in the same models (Figure 13).

The largest displacement value occurring on tooth D6 during tooth loading was observed in B2 models (0.219 mm), with a maximum displacement value of 0.037 mm in B1 and B2 models (Figure 13).

## 4. Discussion

The concept of all-on-four implants has attracted interest in both clinical and experimental studies since it was introduced. It offers an efficient, quick, and cost-effective solution. The patient firstly receives a temporary restoration on just four implants, and after osseointegration, a fixed one, all without extensive surgical procedures [5,6,20,27,28,29].

Materials for fixed full-arch restorations within the all-on-four protocol have evolved from conventional metal–acrylic designs to advanced CAD/CAM-fabricated solutions. High-strength metals, such as milled titanium and cobalt–chromium (Co-Cr) alloys, remain widely used for their durability and manufacturing precision, while zirconia, both monolithic and veneered, has gained prominence for its superior esthetics and strength, despite concerns about its density and stiffness. Hybrid designs, particularly zirconia superstructures supported by titanium bars, have emerged as reliable solutions that combine strength, esthetics, and reduced prosthetic weight. In parallel, digital workflows have enabled the use of alternative materials such as PEEK, PMMA, and fiber-reinforced composites (e.g., Trilor and Trinia), offering advantages in weight reduction, patient comfort, and cost-effectiveness, although with more limited long-term clinical evidence. More recently, graphene has been introduced as a potential framework material, but current clinical data remain scarce [30].

Numerous factors affect the survival of implants and restorations, among which the choice of restorative material holds a significant role. Understanding biomechanical principles is essential in implant prosthodontics. The forces generated in the oral cavity by the masticatory muscles during chewing are transmitted from the occlusal surfaces of the implant-supported fixed dental prostheses (FDPs) to the implants and the surrounding bone. Inadequate loading can lead to bone resorption around the implant (with distal inclined implants being particularly susceptible to this phenomenon) and potential destruction or failure of the prosthetic construction [28]. Therefore, it is crucial to understand how stresses are induced under the influence of occlusal forces. The finite element method remains the most reliable experimental approach for analyzing load distribution within implant-supported prosthetic systems [27].

Our study examined the influence of different loading conditions on stress distribution in the bone surrounding the implant when using various restorative materials. Six models were analyzed, each representing a distinct combination of materials. The distribution of stress within the bone–implant–restoration system was evaluated under both static and dynamic loading conditions.

Available data suggest that the direction of the applied forces is crucial: a horizontal force can generate a greater moment on the implant’s neck, while a vertical force tends to produce a smaller moment [20]. Analysis of the impact of static horizontal and vertical loading in this study revealed a clear link between force direction and material type, affecting the stress distribution within the implant restoration. A static horizontal load of 90 N, applied to the central incisors’ region, caused stress to spread to the anterior segment of the jaw model and restoration. Regardless of the material used, the load distribution was similar, although the stress intensity varied depending on the framework’s modulus of elasticity. For materials with a higher modulus of elasticity, such as ZrO_2_ and CoCr, the stress was concentrated within the framework, primarily on the distal implants, where the highest stress values were recorded. In the cortical bone, a significant stress concentration was observed in the posterior segment, while the trabecular bone experienced more pronounced stresses in the anterior part. The PEEK-made framework exhibited a higher overall stress value but smaller surface concentration zones in the front area. The highest stress concentration was observed on the front implants and abutments at the implant neck, spreading across nearly the entire implant surface. The screws showed stress concentration in the front section, while the crowns experienced stress in the incisor region, particularly where the force is directly applied. Similar findings were reported by Darwish et al. [20], who observed a four-implant model under horizontal force. The authors found the highest stress on the left front implant, while the cortical bone around the right front implant experienced stress on the mesial side in the neck area. The differences between the left and right sides have been attributed to the presence of a metallic superstructure exposed to bending moments [20]. Turker et al. [29] reported that the highest stresses occur precisely near the point of force application, as shown by our study’s results. In the research by Kumari et al. [31], the impact of horizontal loading was analyzed with different angulations of distal implants and cantilever lengths in the “all on four” concept. The highest stresses concentrated around the neck of the distal inclined implants were found to increase with the length of the cantilever. A study by Canay et al. [32] showed that an angulation of 30° during the application of a horizontal load does not cause significant stresses, which is in accordance with our findings, indicating that stresses remain within physiological limits for all analyzed materials.

A vertical load of 150 N, applied bilaterally in the premolar region, caused a different stress distribution pattern. Rigid framework materials bore most of the load, while with PEEK, the stress was more concentrated on the implants. For framework materials with a higher modulus of elasticity, the stress is spread over a larger surface of the framework. In contrast, with PEEK, it was confined to the zones of direct force application. The stress distribution in bone showed a similar pattern: trabecular bone showed larger stress areas. The PEEK model showed the highest stress values in the distal implant’s neck on the mesial side. On the screws, the stress was expressed in the distal part, while the crowns showed a localized concentration of stress in the zones of force action.

The results of Darwish et al. [20], indicate that distally inclined implants are subjected to the highest stresses, especially in the mesial and palatal parts of the bone. Kumari et al. [31] confirm that maximum stresses occur in the cortical bone around the implant neck, with lower values at 30° compared to 40° angulation. These findings are consistent with the results of our study, which examined distal implant angulation at 30°. Several studies [18,19,20,33] likewise demonstrated that the highest stress values occur with distally inclined implants.

Bhering et al. [9] compared prosthetic concepts supported by four and six implants, applying a unilateral load of 150 N on the lateral teeth at a 30° inclination. The authors analyzed the influence of framework material on stress distribution and found that stiffer materials (Zr and CoCr) exhibited higher stress values within the framework compared to softer ones (Ti). However, all stresses remained within the resistance limits of the respective materials. These findings are consistent with our results obtained under a bilateral load of 150 N.

According to literature data, the average biting force is approximately 220 N [34]. Forces acting in the area of the cantilever result in higher framework stresses, especially around the bone near the distal inclined implant in this complex, because they simulate the action of a lever [18]. Research by Darwish et al. [20] indicated that with bilateral vertical molar loading, the maximum stress occurs around the neck of the oblique implant as well as the cortical bone. The results of this study, along with those of Turker et al. [29]. and Sanino et al. [33], confirmed these findings, noting that the stress concentration was nearly twice as high when the distal implant was inclined at 45 degrees compared to 30 degrees. Ersoz and Mumcu [18] applied a vertical force of 200 N unilaterally in the molar region. Authors compared a system with four and six implants and used framework materials such as CoCr, Ti, ZrO, and PEEK. When comparing stress distribution under axial and oblique loading, they found differences related to the modulus of the material’s elasticity. Specifically, PEEK transmitted more stress to implants and bone, unlike solid materials, which distribute stress more evenly to implant abutments and the framework, aligning with the results of this study.

In the bone surrounding the implants, the PEEK framework generated approximately twice the stress compared to other materials. The implant–abutment complex was most intensively loaded in the PEEK model, particularly in the distal region, with a similar distribution observed on the denture screws. A recent study reported that under both observed loading modes (axial and oblique), the highest stress on abutment screws was concentrated in the cervical region, which the authors attributed to the limited dissipation of forces when the load is applied at the terminal denture position [18]. Dayan et al. [11] likewise confirmed that with more elastic materials, such as PEEK and PEKK (polyetherketoneketone), stress tends to concentrate in the cervical region of the screw. The crowns of the PEEK model in this study showed a higher surface distribution and stress intensity.

In our study, we used zirconia ceramic and a composite as the veneer materials to assess their effects on stress distribution. The study demonstrated that ceramic veneers show higher stress levels across all load cases and models compared to composite veneers. The stress values on the veneers are greater in the B model for all load cases. Our results indicate that the veneer material does not influence stress distribution on the implant and surrounding bone. Conversely, Haroun et al. considered the veneering material in their study and concluded that combining superstructure material with framework materials can serve as a stress dampener. Haroun et al. observed lower stress levels in the bone when using PEEK instead of titanium frameworks [8]. These differences may stem from variations in study design and methodological approaches.

Limited research has been conducted on dynamic loading. Geramizadeh et al. [22], who examined static and dynamic loading of implants as well as the screw thread material fatigue, found that dynamic loading causes 5 to 10% higher VM stress on the bone surrounding the implant compared to static loading. Kabayashi et al. [35] applied a 100 N load in three 15 min sessions, simulating three meals. Authors indicate that there is higher VM stress on the bone around the distal implant, which is in line with our results, despite differences in methodology.

In this study, dynamic loading was simulated by a 150 N force that varied in terms of application point, direction, and duration throughout the masticatory cycle. The loads were applied to certain teeth within the internal occlusal field. The peak intensity was observed at 0.17 s, after which the system gradually transitioned into a state of stabilization, as documented in the analysis.

During dynamic loading on the premolar (DO1), the distribution pattern at moments of maximum stress depended on the framework material. In the PEEK models (B1 and B2), stress was concentrated on a smaller surface, especially in the region of force application. The highest values were recorded on implants, cortical bone, abutments, and screws, while trabecular bone showed the lowest levels. In the CoCr models (A1 and A2), the stress fields were considerably wider, spreading across the entire structure. Maximum stress occurred within the framework itself, particularly in model A2 with composite crowns. The distribution followed a descending order: implants, abutments, cortical bone, trabecular bone, and screws. In the ZrO models (C1 and C2), stress fields were more evenly distributed over a larger portion of the structure. The highest stresses were concentrated within the framework, followed by the crowns, the implant–abutment complex, cortical bone, and screws, with the lowest values again observed in trabecular bone.

In the case of molar loading, all stress values were increased, while the distribution pattern remained identical to that of the premolars.

The study by Liu et al. [25], which investigated the effects of dynamic loading in all-on-four concepts, demonstrated that the highest stress occurs at the neck of oblique implants, with distal implants generally exhibiting higher values than mesial ones. The authors also analyzed various positions of the anterior implants and observed that stress distribution is more favorable when these implants are placed in the canine fossa region. Regarding the surrounding bone, the highest stress values were recorded at the margin of the cortical bone around the distal implant, which is consistent with the findings of this study and others [36,37]. These results support the observation that bone resorption tends to develop in the cervical cortical region around distal implants, most likely due to the increased stress concentration at the implant neck.

The results of the study by Sahin Hazir et al. [38], consistent with findings from other authors [9,10,11,12,13,14,15,39] indicate that frameworks with a higher modulus of elasticity are more resistant to deformation but exhibit higher stress concentrations. However, these stresses do not necessarily lead to clinical complications, as stiffer frameworks are associated with a corresponding reduction in VM stress in the abutments. This suggests that the increased rigidity of the framework may decrease the risk of mechanical overload.

Under dynamic loading at positions L5 and L6, the maximum stress values within the structural components were compared to the threshold values from the S–N diagram (fatigue curves) to assess the material’s resistance to cyclic loading.

The study by Darwish et al. [20], which investigated static loading and material fatigue in both conventional and implant-supported models, reported higher stress values under fatigue loading conditions. In our study, cyclic loading of 10^9^ cycles indicated the potential for fatigue-related fracture of the cortical bone in the region of the terminal implants in models B1 and B2 with PEEK frameworks. It is important to emphasize that fatigue failure does not necessarily imply complete structural failure. In this case, cortical bone fracture remains localized around the implant neck, after which the load transfer is redistributed to the trabecular bone, which demonstrates more favorable behavior under load when combined with PEEK frameworks. Cortical bone exhibits higher fatigue values compared to trabecular bone in material fatigue testing. The maximum stress capacity of cortical bone can be attributed to its biological structure, as its hard, dense composition contrasts with the porous and brittle nature of trabecular bone. Regarding physiological limits (ultimate bone strength), all stress values in cortical bone are below the conventional endurance limit of 185.3 MPa. Ultimate bone strength for trabecular bone is 5 MPa [20].

Materials with a higher modulus of elasticity demonstrated greater safety factors (SF) and improved resistance to cyclic loading. Displacement represents the deviation of the material from its reference point in the coordinate system. Excessive movement of the free end, i.e., the cantilever, in implant-supported fixed restorations can compromise masticatory efficiency and threaten long-term stability. Previous studies have shown that rigid frameworks reduce implant stress and consequently minimize denture displacement. In the study by Hazir et al. [38], displacement of PEEK frameworks reached as much as 0,35 mm. Similarly, Chen et al. [1] compared three framework materials (CoCr, PEEK, and Ti) and reported the largest displacements with PEEK, particularly at the cantilever, which may predispose to polymer framework collapse. Bhering et al. [9] also concluded that materials with a higher modulus of elasticity are more resistant to displacement and deformation, although they generate higher stress values, a finding consistent with other reports [11,13]. The use of stiff framework materials is therefore recommended to prevent mechanical damage to the system, as they are associated with reduced stress in cortical bone, implants, abutments, and screws, as well as lower overall displacement.

In this study, models B1 and B2 exhibited significantly greater structural displacement compared to models A1, A2, C1, and C2. This finding indicates reduced stability of the PEEK framework under dynamic conditions and further supports the unfavorable assessment of its mechanical performance during long-term function.

The analysis of the displacement results shows significant differences in the mechanical behavior of different models of implant structures under dynamic load, depending on the place of force application (D5 or D6), the direction of displacement (horizontal or vertical), as well as the materials used.

In the horizontal direction, the most pronounced movement was registered with models B2 and B1, which indicates a lower rigidity of these structures. In particular, the displacement of −0.681 mm in model B1 (D6) represents a potential risk for the long-term stability of the structure and may affect the distribution of forces in the periodontal area. This indicates an increased risk of screw loosening or fracture, the most common complications of screw-retained implant-supported restorations. For patients, this results in decreased chewing efficiency because of poorer retention [5,40]. On the other hand, models A1 and A2 showed the least displacement in both load cases, which may indicate greater stability and better ability to absorb dynamic forces.

The vertical direction exhibited smaller absolute displacement values than the horizontal direction, which can be attributed to how the construction is fixed and its contact with the alveolar ridge. Models B1 and B2 again show the most movement, especially when force is applied to tooth D6 (−0.219 mm in B2), which may indicate a more flexible material or a structural flaw in that area.

It is noteworthy that models from group C (C1 and C2) demonstrated consistently lower displacement values in both directions, suggesting a favorable balance between rigidity and elasticity of the material. These findings may have clinical relevance, particularly in the selection of frameworks for patients with higher functional demands or in cases involving the replacement of multiple teeth.

Bhering et al. [9], Chen et al. [1], and Hazir et al. [38] likewise concluded that PEEK frameworks exhibit greater displacement. Hazir further emphasized that materials with a higher modulus of elasticity generally show reduced displacement while retaining more stress within the framework itself, which is consistent with the findings of our study.

### Clinical Significance

Although combinations with PEEK exhibit greater flexibility, they do not provide adequate fatigue resistance of the cortical bone, particularly under long-term cyclic loading. The observed fractures and displacements may adversely affect implant stability, promote bone resorption, and compromise the long-term functionality of the restoration. Within the limitations of the present study, it should be emphasized that FEA, although providing valuable biomechanical insights, does not account for biological adaptation, patient-specific variability, or clinical handling factors that may influence the long-term performance of restorative materials. Our findings suggest that PEEK exhibits inferior fatigue behavior. In contrast, materials with a higher modulus of elasticity demonstrate more favorable stress distribution and an increased safety factor. The oral environment represents a highly complex biomechanical system in which loading conditions are governed by multiple factors, including muscle activity, patient age, general health status, antagonist characteristics, material of restoration, as well as manufacturing techniques and their quality. Although experimental studies cannot replace well-designed prospective clinical trials, they offer a strong scientific basis for generating hypotheses and can serve as a helpful guide for future clinical decisions and research directions. At the same time, it is important to highlight the importance of well-designed clinical studies with sound methodology, adequate sample sizes, and long-term follow-up to ensure clinical reliability. Combining numerical simulations with clinical follow-ups would further enhance our understanding of the clinical relevance of these findings.

Every experimental method, including the finite element analysis, has its limitations. The precision, fidelity, and computational power of the model reconstruction significantly influence the accuracy of FEA results [18]. Despite the insights provided by this study, several limitations must be acknowledged. The results of our study represent single simulations without measures of variability or uncertainty. The shortcomings of this study are with respect to individual structural components. All interfaces (bone–implant, implant–abutment, abutment–framework, framework–crown) are modeled as perfectly bonded (tied contacts). This eliminates micromovements, fretting, and non-linear contact behavior that occur clinically, potentially underestimating stress concentrations at interfaces. Also, bone tissue is represented as homogeneously isotropic, but it is naturally anisotropic. Complete osseointegration in real situations does not exist; however, in these studies, it is mostly presented as 100% contact. Bone–implant contact of 100% is clinically unrealistic, particularly in D3 bone quality. This may overestimate load transfer efficiency and underestimate implant stress. It should be emphasized that finite element analysis, although invaluable for analyzing forces that cannot be measured in a living system, provides a valuable guide for future clinical trials. The strength of this study lies in the use of dynamic loading, which more realistically represents mastication and should be more employed in experimental studies. This study compares the results of static and dynamic loading. Also, the value of the study is reflected in the presentation of the stress distribution across all components of the complex (implant, abutment, screw, cortical bone, cancellous bone, framework, and crowns). The study also examined the behavior of restorations made of different materials under varying loading conditions, as well as material fatigue and restoration displacement. Greater study validity could be achieved by using a CBCT scan of the patient’s jaw, whereas a scan of the upper jaw model was used in this research.

## 5. Conclusions

According to the results of this study, stress distribution within the bone–implant–superstructure system is influenced by several factors, including the characteristics of the applied force—its direction, point of application, and intensity. The modulus of elasticity of the framework material affects both the magnitude and the spatial extent of stress in the surrounding bone, thereby determining the overall load distribution within the system. Within the limitations of the present study design, the veneering material did not significantly influence load distribution across the remaining components of the system. Dynamic loading produces higher stress intensities and offers a more realistic representation of the biomechanical conditions that occur during mastication. Furthermore, the presence of a cantilever element, particularly when the load is applied to it, exerts a more pronounced effect on stress magnitude than any other examined factor.

While finite element analysis provides valuable insights into the biomechanical behavior of implant-supported prostheses, further long-term clinical studies are necessary to validate these findings.

## Figures and Tables

**Figure 7:**
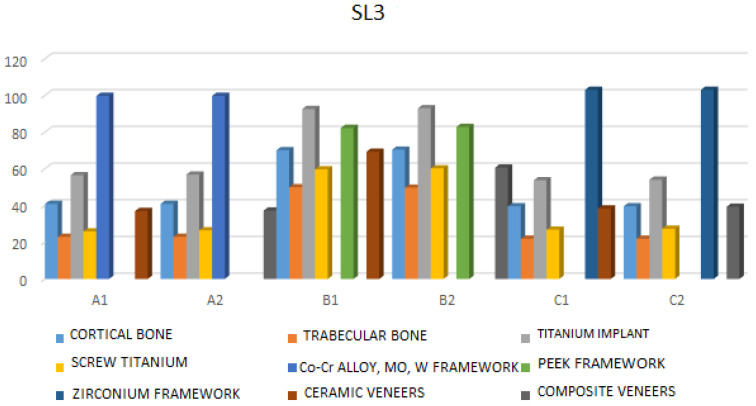
Maximum stress values under bilateral vertical static loadings (SL3) (F = 200 N, teeth: the first right molar, the first left molar).

**Table 1:** The models used in static and dynamic analysis.

Model	Implants and Abutments	Screws	Framework	Veneering Materials
A1	Titanium	Titanium	Co-Cr alloy *	Ceramics
A2				Composite
B1			PEEK **	Ceramics
B2				Composite
C1			Zirconium	Ceramics
C2				Composite

* Cobalt chromium alloy; ** Polyetheretherketone.

**Table 2:** Number of nodes and elements adopted for the models.

Set	Number of Nodes	Number of Elements
Cortical bone	329,209	188,891
Cancellous bone	433,480	289,693
Implants and abutments	73,581	47,297
Prosthetic screw	8486	4922
Framework	341,625	225,936
Veneers	274,987	174,606
Total	1,342,629	931,345

**Table 3:** Mechanical properties of materials used in finite element analysis.

Structure	Young’s Modulus (GPa)	Poisson’s Ratio	Density (g/cm^3^)
Cancellous bone	1.37	0.30	0.90
Cortical bone	13.7	0.30	1.85
Titanium grade 5	110.0	0.35	4.50
Co-Cr alloy *	208.0	0.31	8.90
PEEK **	4.1	0.36	1.30
Zirconia	269.0	0.25	5.86
Composite (Gradia)	50.0	0.30	1.40
Feldspathic ceramic	82.8	0.35	2.45

* Cobalt chromium alloy; ** Polyetheretherketone.

## Data Availability

The original contributions presented in the study are included in the article; further inquiries can be directed to the corresponding author.

## Supplementary Materials

The following supporting information can be downloaded at: https://www.mdpi.com/article/10.3390/jfb17050238/s1, Figure S1: Effective stress field in the model for the case of loading SL1—Section along the axis of the implant and screw (MPa); Figure S2: VM stress peaks on cancelous bone; Figure S3: VM stress peaks on prostetic screws; Figure S4: VM stress peaks on crowns; Figure S5: VM stress peaks on implant-abatment complex; Figure S6: VM stress peaks on cortical bone; Figure S7: VM stress peaks on framework; Figure S8: Effective stress field in the model for the case of loading SL2—Section along the axis of the implant and screw (MPa); Figure S9: Effective stress field in the model for the case of loading SL3—Section along the axis of the implant and screw (MPa); Figure S10: Effective stress field in crowns for the case of dynamic loading DL2 at time 0.17s Section along the axis of the implant and screw (MPa).

## Abbreviations

The following abbreviations are used in this manuscript:

SL	Static load
DL	Dynamic load
PEEK	Polyetheretherketone
CoCr	Cobalt–Chromium
Zr	Zirconia
