# Supplementary figures and images for: Influence of Framework Material on Stress, Fatigue, and Stability of “All-on-Four” System Components—Biomechanical Evaluation with Finite Element Analysis
Source: J Funct Biomater. 2026 May 8;17(5):238. doi: 10.3390/jfb17050238 (PMC13208333; doi:10.3390/jfb17050238)

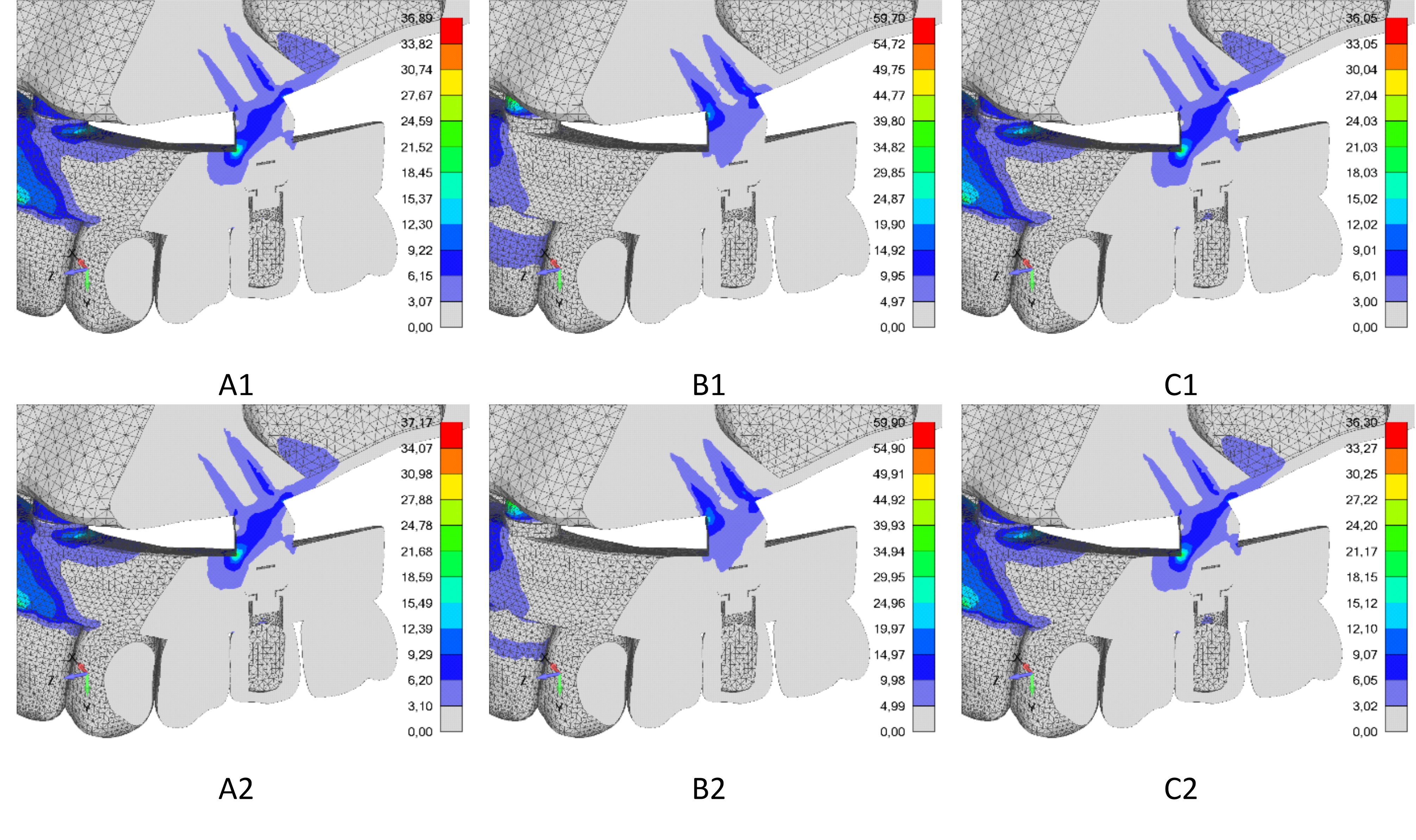

Supplement: Supplementary file 1 [file jfb-17-00238-s001.zip › Supplementary Figure S1 Effective stress field in the model for the case of loading SL1 - Section along the axis of the implant and screw (MPa).jpg]

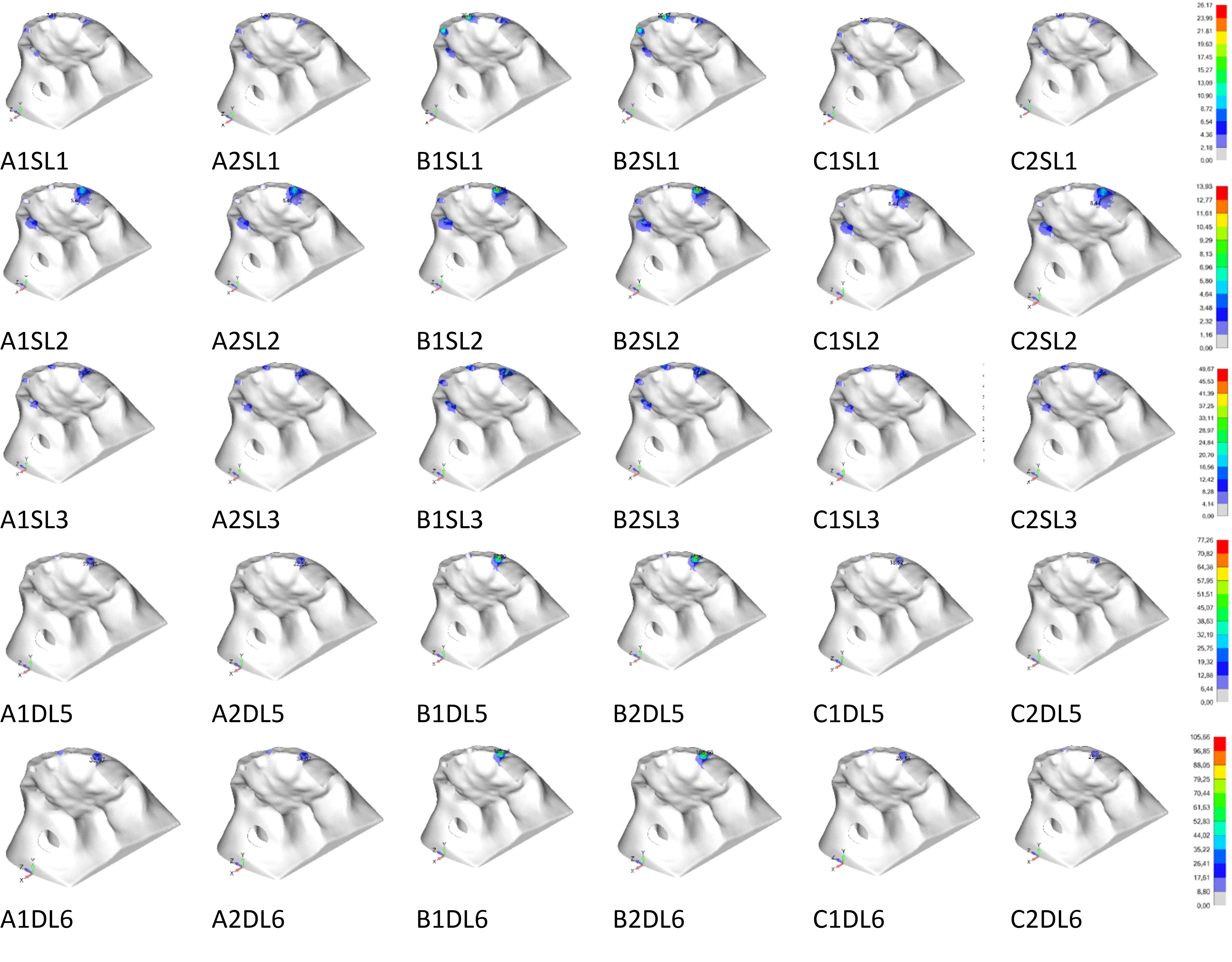

Supplement: Supplementary file 1 [file jfb-17-00238-s001.zip › Supplementary Figure S2 VM stress peaks on cancelous bone.jpg]

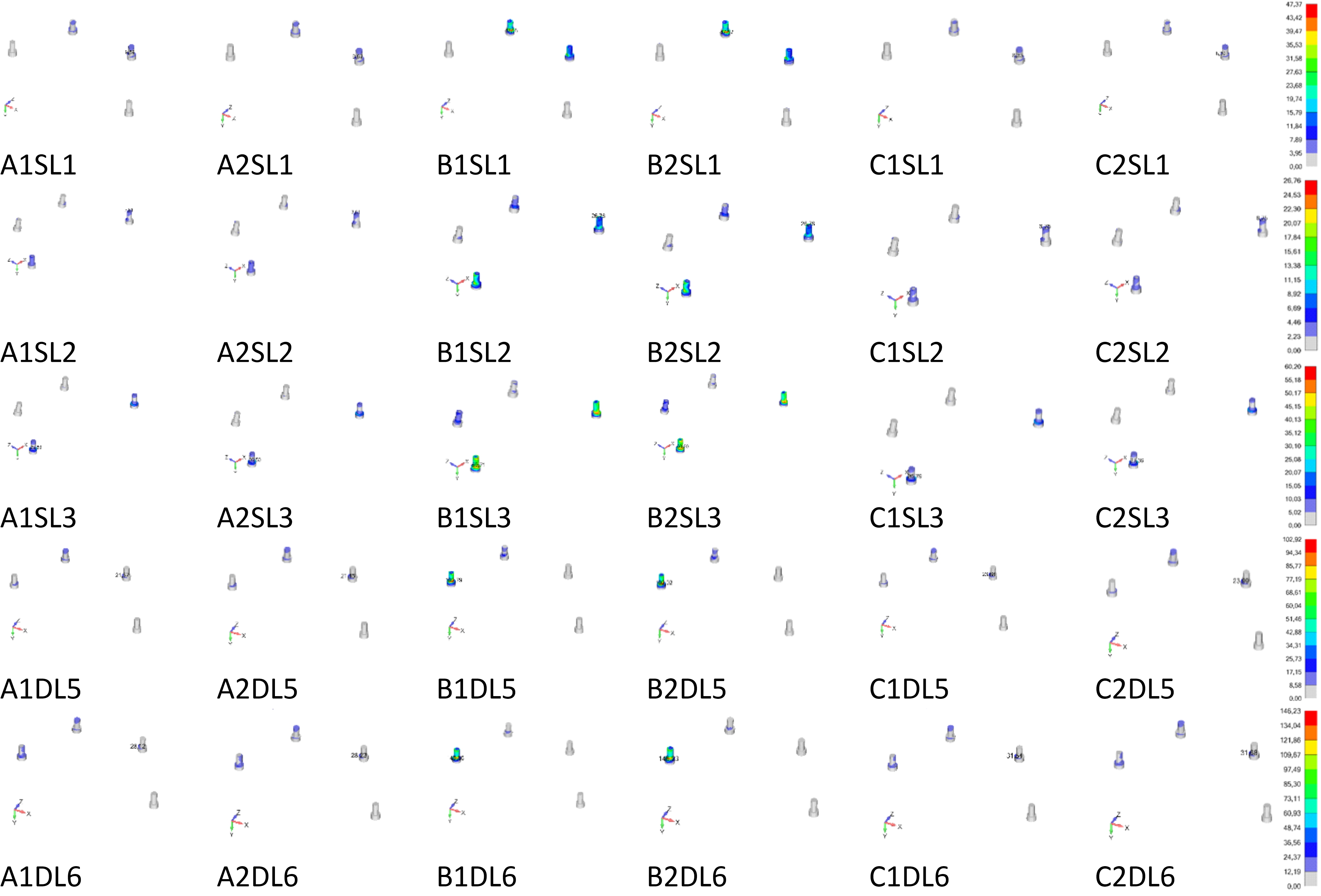

Supplement: Supplementary file 1 [file jfb-17-00238-s001.zip › Supplementary Figure S3 VM stress peaks on prostetic screws.jpg]

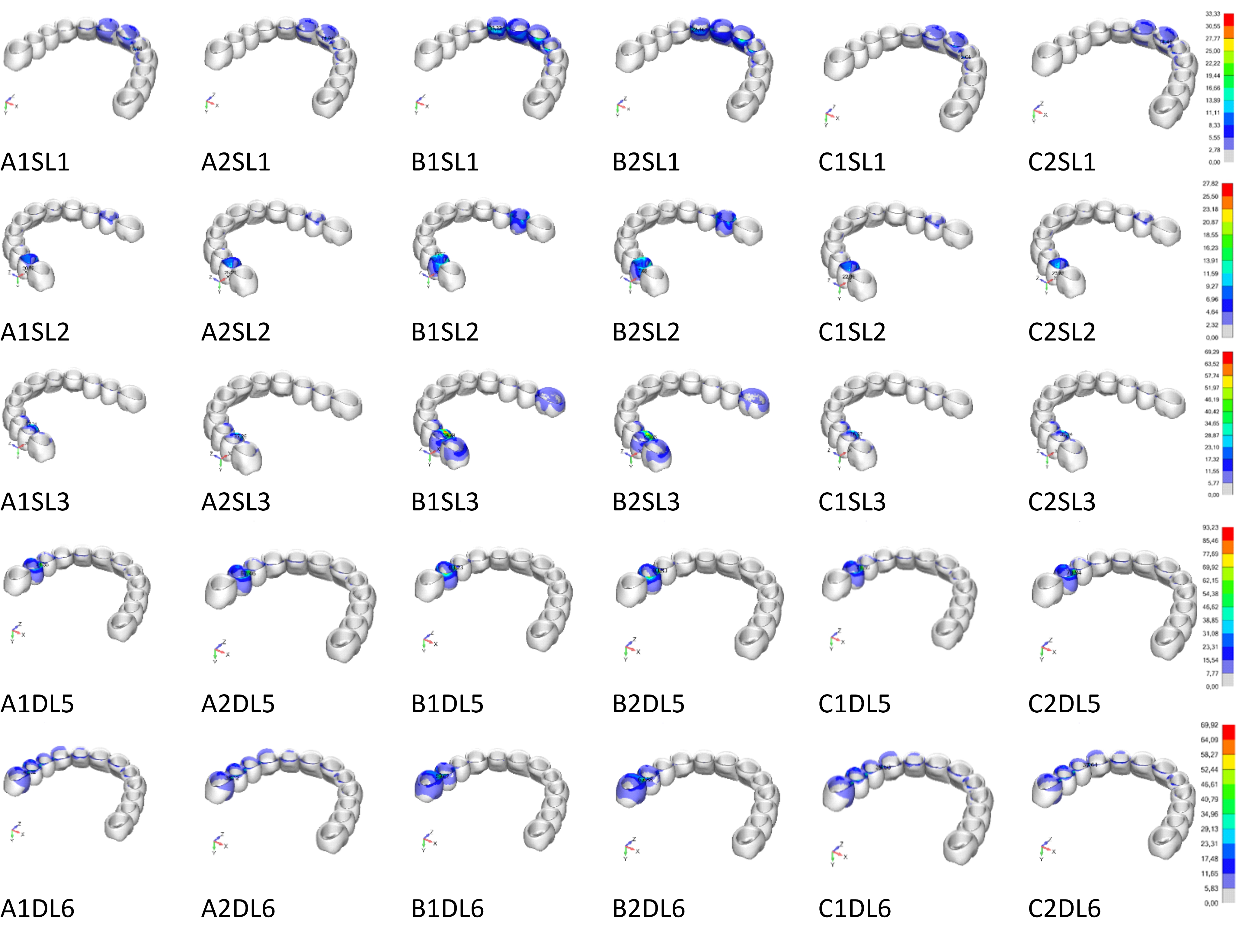

Supplement: Supplementary file 1 [file jfb-17-00238-s001.zip › Supplementary Figure S4 VM stress peaks on crowns.jpg]

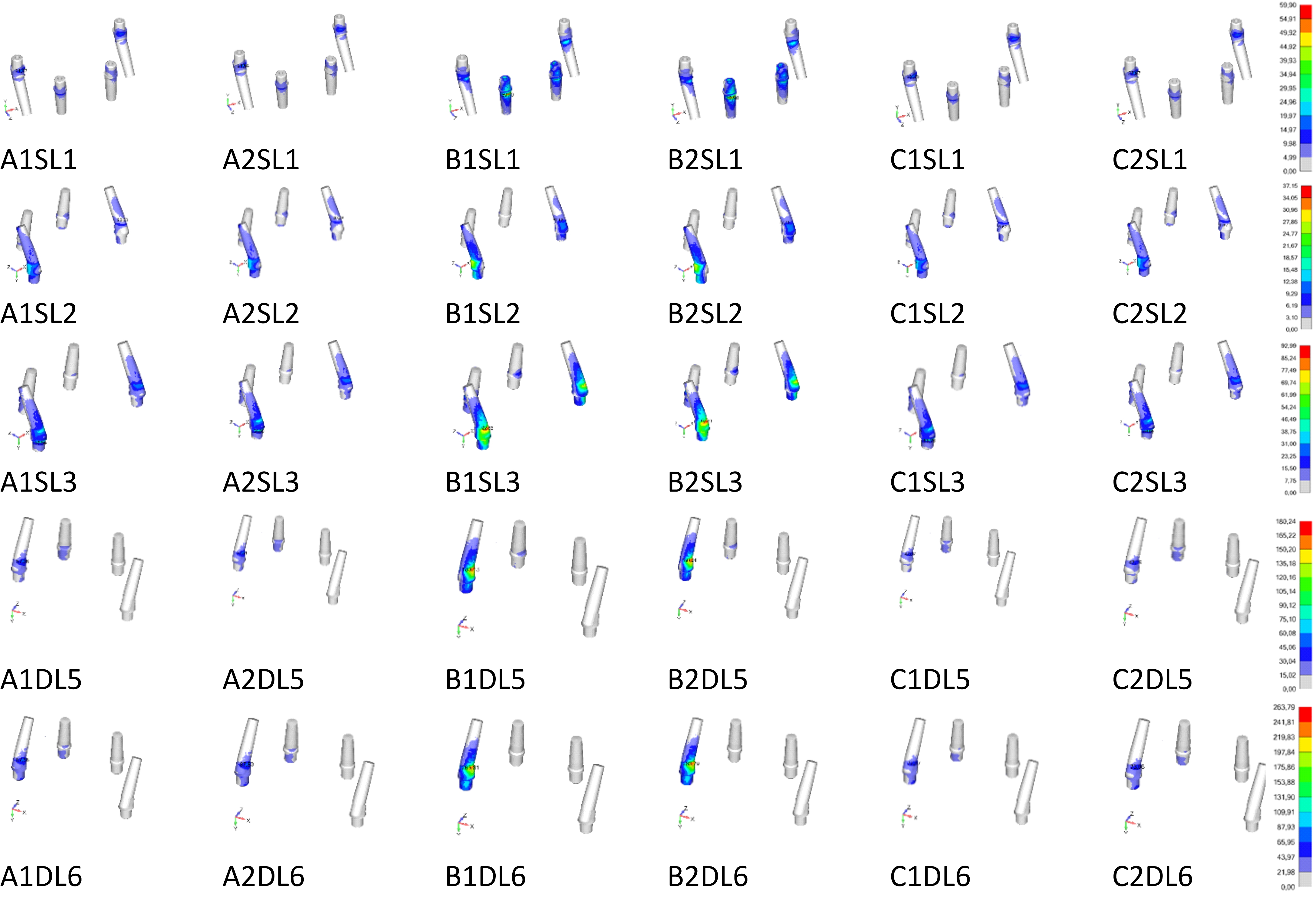

Supplement: Supplementary file 1 [file jfb-17-00238-s001.zip › Supplementary Figure S5 VM stress peaks on implant-abatment complex.jpg]

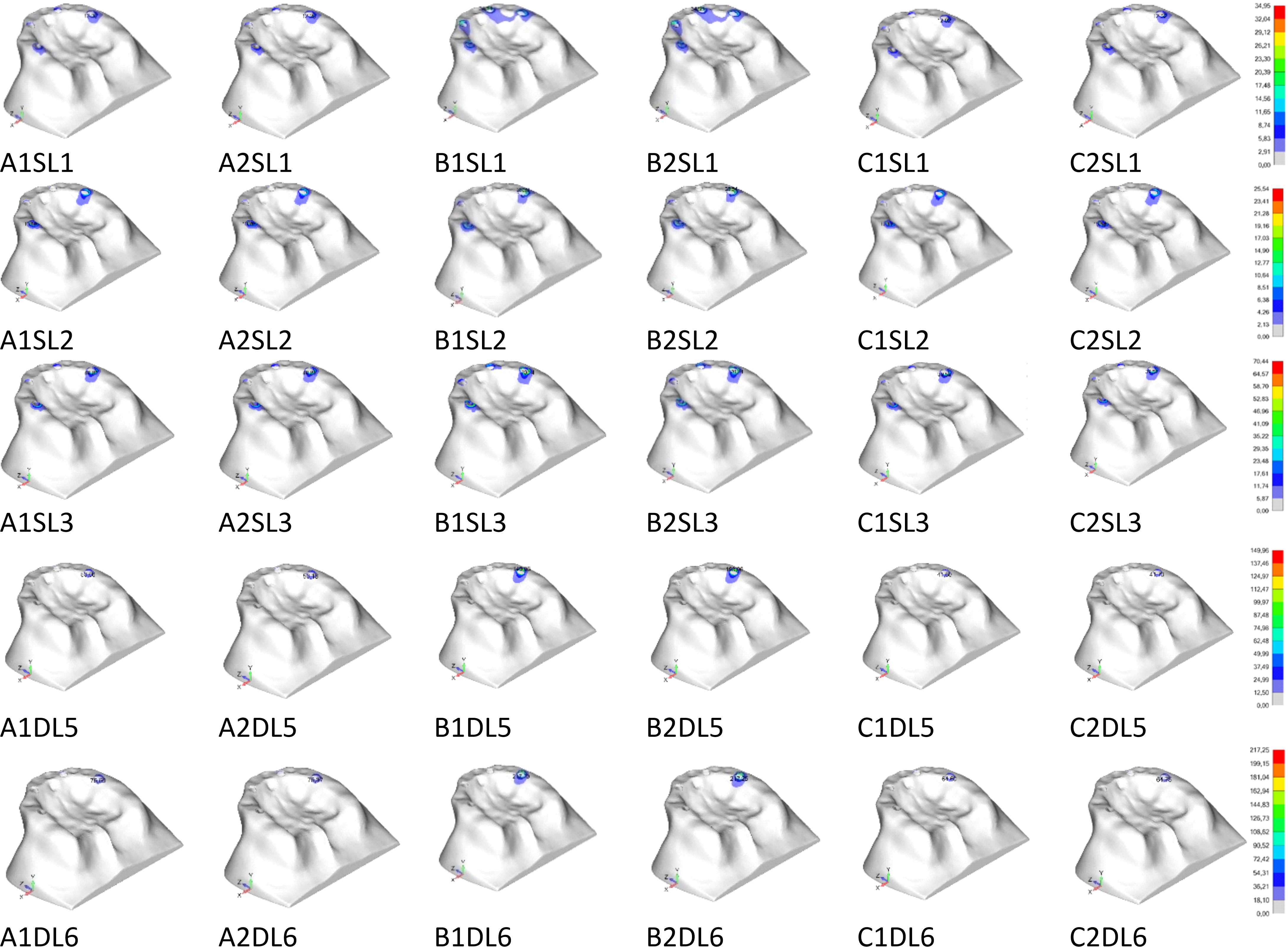

Supplement: Supplementary file 1 [file jfb-17-00238-s001.zip › Supplementary Figure S6 VM stress peaks on cortical bone.jpg]

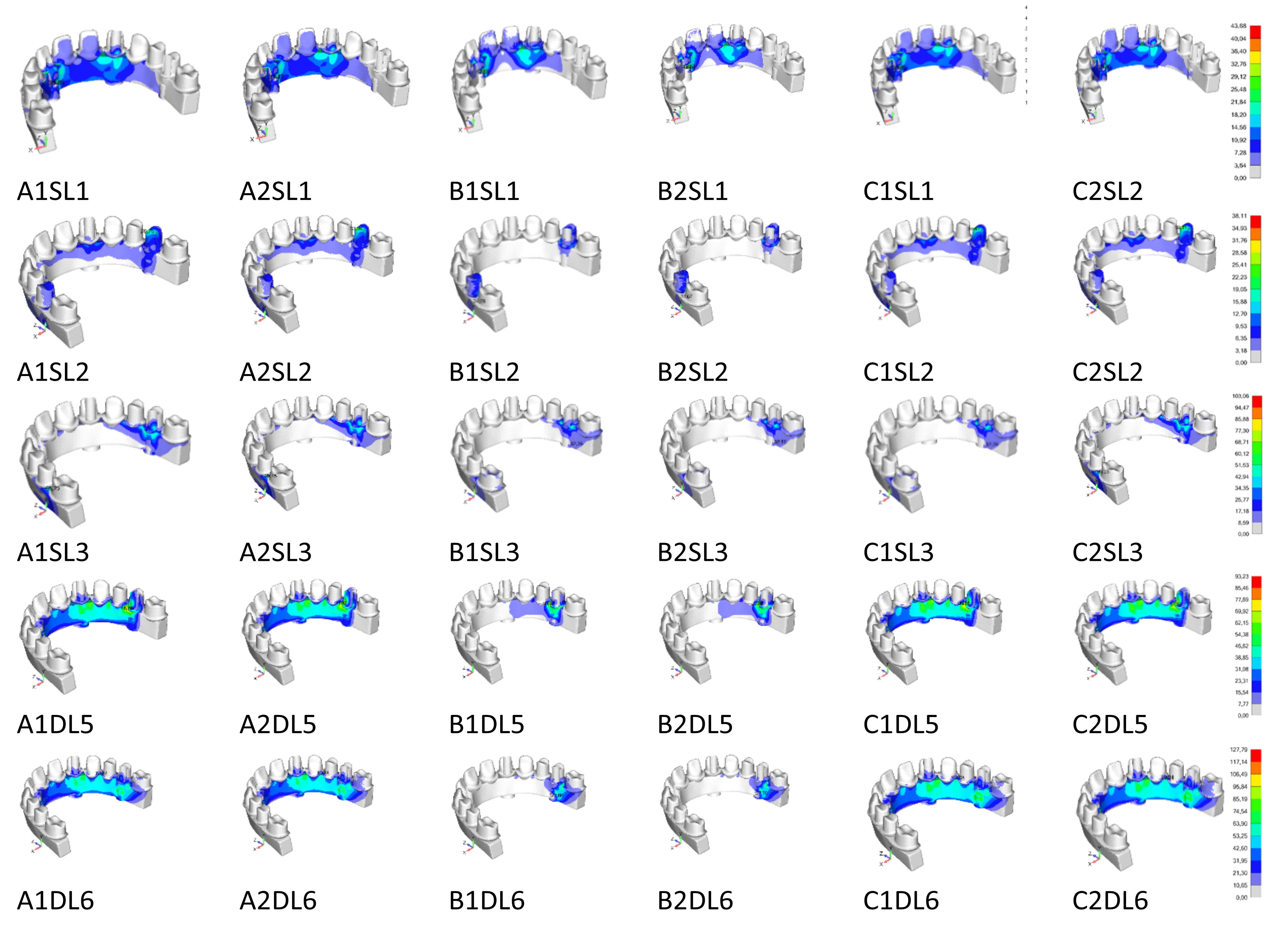

Supplement: Supplementary file 1 [file jfb-17-00238-s001.zip › Supplementary Figure S7 VM stress peaks on framework.jpg]

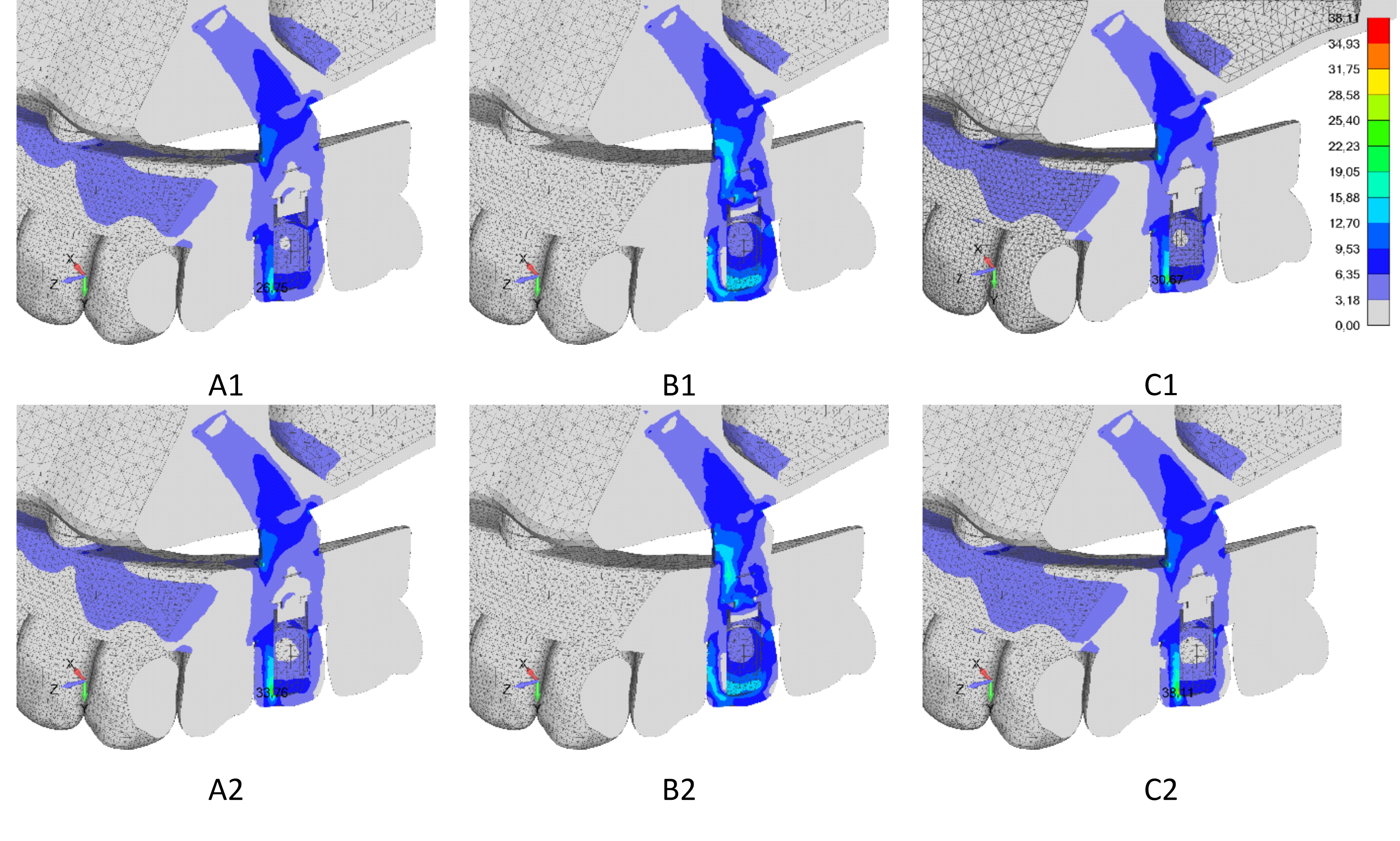

Supplement: Supplementary file 1 [file jfb-17-00238-s001.zip › Supplementary Figure S8 Effective stress field in the model for the case of loading SL2 - Section along the axis of the implant and screw (MPa).jpg]

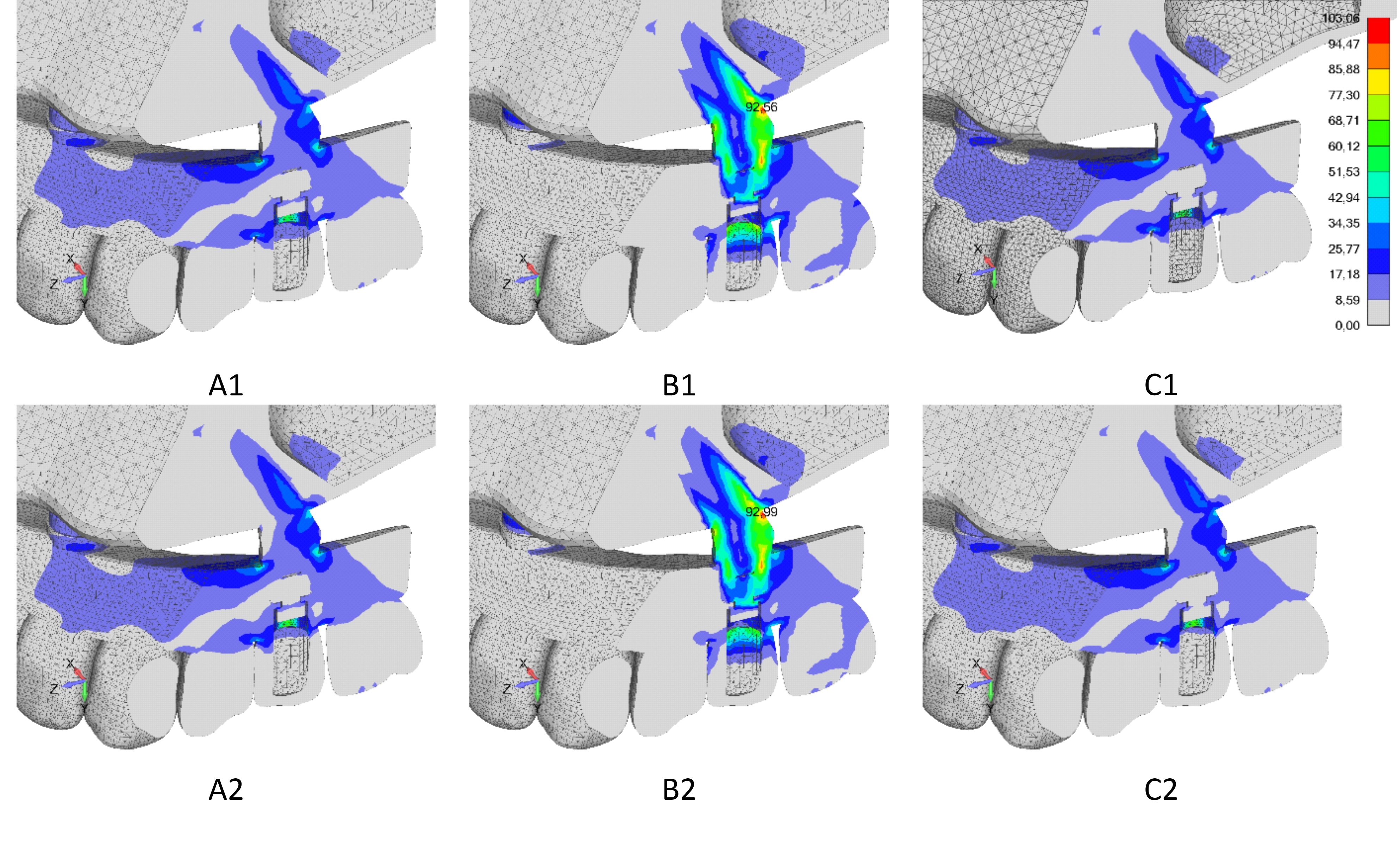

Supplement: Supplementary file 1 [file jfb-17-00238-s001.zip › Supplementary Figure S9 Effective stress field in the model for the case of loading SL3 - Section along the axis of the implant and screw (MPa).jpg]

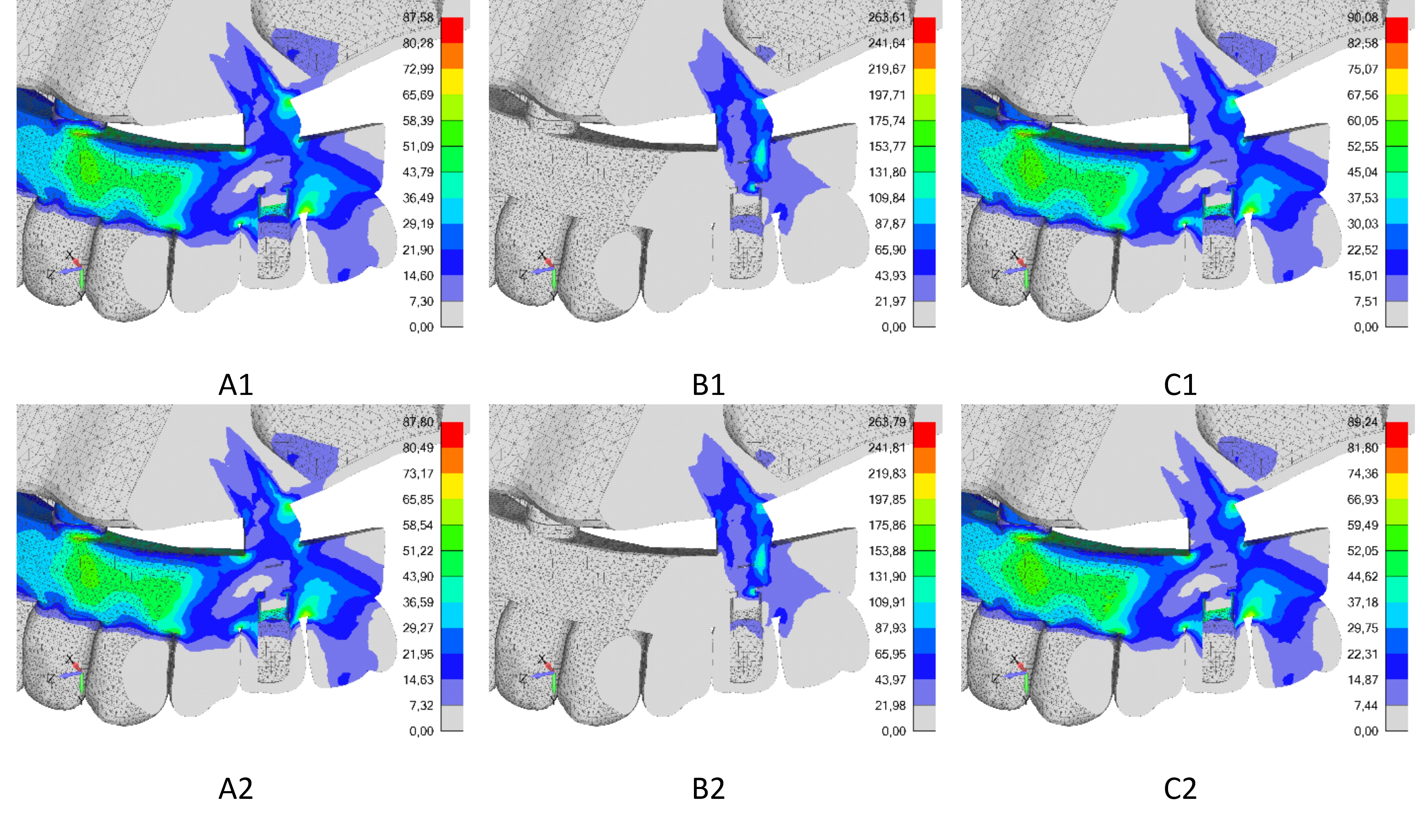

Supplement: Supplementary file 1 [file jfb-17-00238-s001.zip › Supplementary Figure S10 Effective stress field in crowns for the case of dynamic loading DL2 at time 0.17s Section along the axis of the implant and screw (MPa).jpg]
